# Advanced maternal age and adverse pregnancy outcomes: A systematic review and meta-analysis

**DOI:** 10.1371/journal.pone.0186287

**Published:** 2017-10-17

**Authors:** Samantha C. Lean, Hayley Derricott, Rebecca L. Jones, Alexander E. P. Heazell

**Affiliations:** 1 Maternal and Fetal Health Research Centre, Division of Developmental Biology and Medicine, Faculty of Biology, Medicine and Health, University of Manchester, Manchester, United Kingdom; 2 St. Mary's Hospital, Central Manchester University Hospitals NHS Foundation Trust, Manchester Academic Health Science Centre, Manchester, United Kingdom; The Hospital for Sick Children, CANADA

## Abstract

**Background:**

Advanced maternal age (AMA; ≥35 years) is an increasing trend and is reported to be associated with various pregnancy complications.

**Objective:**

To determine the risk of stillbirth and other adverse pregnancy outcomes in women of AMA.

**Search strategy:**

Embase, Medline (Ovid), Cochrane Database of Systematic Reviews, ClinicalTrials.gov, LILACS and conference proceedings were searched from ≥2000.

**Selection criteria:**

Cohort and case-control studies reporting data on one or more co-primary outcomes (stillbirth or fetal growth restriction (FGR)) and/or secondary outcomes in mothers ≥35 years and <35 years.

**Data collection and analysis:**

The effect of age on pregnancy outcome was investigated by random effects meta-analysis and meta-regression. Stillbirth rates were correlated to rates of maternal diabetes, obesity, hypertension and use of assisted reproductive therapies (ART).

**Main results:**

Out of 1940 identified titles; 63 cohort studies and 12 case-control studies were included in the meta-analysis. AMA increased the risk of stillbirth (OR 1.75, 95%CI 1.62 to 1.89) with a population attributable risk of 4.7%. Similar trends were seen for risks of FGR, neonatal death, NICU unit admission restriction and GDM. The relationship between AMA and stillbirth was not related to maternal morbidity or ART.

**Conclusions:**

Stillbirth risk increases with increasing maternal age. This is not wholly explained by maternal co-morbidities and use of ART. We propose that placental dysfunction may mediate adverse pregnancy outcome in AMA. Further prospective studies are needed to directly test this hypothesis.

## Introduction

Advanced maternal age (AMA) is defined as childbearing in a woman over 35 years of age and is a growing trend within high-income countries [1] In 2013, 20% of births in England and Wales were to women aged 35 years or over and 4% to women ≥40 years compared to 6% and 1% respectively in 1980 [2] This trend is most commonly attributed to older primigravid women who delay childbearing by lifestyle choice or due to underlying subfertility, but also includes multiparous women continuing childbearing [3] Women in both groups have benefited from advancements in assisted reproductive technologies (ART). Although changes in social-economic circumstances and developments in ART have driven a shift towards childbearing later in life, this new trend potentially creates a clinical risk. AMA is reported to be associated with a range of pregnancy complications including: fetal growth restriction (FGR), preeclampsia (PE), placental abruption, pre-term birth (PTB) and stillbirth by a series of epidemiological studies [4–8] and importantly these increased risks appeared to be independent of maternal co-morbidities [9–11] Furthermore, systematic reviews and meta-analyses have demonstrated that AMA is associated with an increased risk of Caesarean birth [12] and that AMA is a risk factor for stillbirth [13] However, these latter studies have been limited by a lack of data regarding the causation of stillbirth in AMA pregnancies and have considered stillbirth in isolation. A structured review of pregnancy risks in women ≥45 years found increased rates of pre-existing hypertension and pregnancy complications, such as gestational diabetes mellitus (GDM), gestational hypertension and PE, all of which may predispose to stillbirth [14] Presently, the majority of stillbirths in high-income countries are related to placental dysfunction [15] Therefore, further studies are needed to examine the relationship between AMA and adverse pregnancy outcomes that are related to placental dysfunction to consider the underlying cause(s) for the reported increased risk of stillbirth in women of AMA.

### Objectives

This systematic review’s objective is primarily to examine the strength of association between AMA and stillbirth and, secondarily, whether AMA is also associated with other pregnancy complications suggestive of placental dysfunction (e.g. FGR) that may explain this relationship. Furthermore, this review will evaluate the impact of maternal co-morbidities (obesity, diabetes and hypertension) and use of assisted reproductive techniques (ART) on stillbirth rates in AMA.

## Methods

### Information sources, search strategy and eligibility criteria

A systematic review and meta-analysis were conducted in accordance with the Preferred Reporting Items for Systematic Reviews and Meta-Analyses (PRISMA) guidelines and Meta-Analysis of Observational Studies in Epidemiology Group (MOOSE) criteria [16] Literature searches were conducted in MEDLINE (Ovid), EMBASE, ClinicalTrials.gov and LILACS and the Cochrane Database of Systematic Reviews using the search terms “maternal age”, “advanced”, “pregnancy” and “outcome” combined. The search was restricted to publication year ≥2000, full text and English language articles (including foreign language articles pre-translated by publisher). Foreign language articles were not included as translation services were unavailable. The search strategy can be found in the appendix. In addition, reference lists from included original papers and review articles were searched to identify further relevant studies. Additional searches were conducted to identify published perinatal mortality reviews (e.g. Confidential Enquiries into Stillbirths, National Review of Perinatal Mortality).

### Data extraction

Observational studies using case control and cohort designs that reported one or more of the pre-specified primary and/or secondary outcomes for maternal age <35 (control) and ≥35 years (AMA) populations were included. Primary outcomes were stillbirth (according to individual study gestational age cut off) and fetal growth restriction (FGR) defined as birthweight below 5^th^ centile adjusted for gestational age [17] Secondary outcomes were neonatal death (NND), small for gestation age (SGA; defined as a birthweight below 10^th^ centile adjusted for gestational age or related definitions specified by authors), neonatal intensive care unit (NICU) admissions and neonatal acidosis (umbilical artery pH <7.0–7.2), preeclampsia (blood pressure ≥140/90 with significant proteinuria or as classified by authors where definition was not provided), placental abruption (classified by authors), preterm birth (PTB) <37 weeks gestation and gestational diabetes mellitus (GDM). Where authors stated different definitions of outcomes, data were re-classified in line with definitions stated (e.g. if authors defined FGR as <10^th^ Centile this was re-classified as SGA in these analyses). Where definitions of classifications were not stated, authors’ classifications were accepted. Where possible, extracted data was sub-divided by parity (primiparous and multiparous mothers). Where reported, data regarding the frequency of maternal co-morbidities (obesity, hypertension and diabetes) and use of ART were extracted.

Duplicate studies were removed and the papers were excluded if they: were case reports, were restricted to multiple pregnancies or did not separate data from multiple pregnancies from singletons, primarily reported the success rates of assisted reproductive technologies or pre-existing medical conditions as a primary outcome, focused on chromosomal abnormalities or substance abuse, relevant data could not be extracted or were review papers. Reports on rising Cesarean section rates with AMA were not included as a systematic review was recently conducted on this topic [12] The initial search was conducted by one investigator (SL) and validated by a secondary conductor (HD) to ensure accuracy of search and application of exclusion criteria.

### Risk of bias assessment

Due to methodological issues regarding quality assessment of observational studies in epidemiology we assessed the quality of studies in eight specific domains that were relevant to the studies in question developed from Sanderson *et al*. [18] specifically to address issues with observational studies. Quality was assessed by risk of bias factors including: selection bias, measurement of outcome bias, assessor bias, completeness of data and data reporting, controlling for confounders, appropriateness of statistical analysis, and conflicts of interest. Quality assessment was performed by two independent investigators (SL and AH) and discrepancies were discussed with a third investigator (HD). Risk of bias was assessed for each of the domains as low, high or unclear using pre-specified rules [18] Data from included studies were extracted by two independent investigators (SL and AH) using a standard extraction form.

### Data synthesis

Meta-analysis was conducted using STATA (Version 13, StataCorp, Texas, USA) using the metan, meta-regression and meta-bias commands [19] Random effects meta-analysis was performed in view of the anticipated heterogeneity between the studies. Heterogeneity was quantified using Cochran’s χ^2^ test to generate I^2^ statistic as a percentage of variability. Heterogeneity was classified as low (I^2^ = 0–40%), moderate (I^2^ = 30–60%), high (I^2^ = 50–90%) or severe (I^2^ = 80–100%) [20] Meta-regression was undertaken to test the effect of maternal age as a linear variable on studies where the median age was reported or could be calculated as the median value of the stated age group.

A sensitivity analysis was conducted to explore heterogeneity; Forest plots were constructed to allow differences in maternal age groups, geographical location and year of study on each outcome. In addition, funnel plots and contour enhanced funnel plots were created to test for publication bias and small study effects quantified by Harbord’s test. Population attributable risk (PAR) was calculated for the proportion of stillbirths associated with AMA based on the prevalence of AMA and the relative risk of stillbirth due AMA. Spearman rank-order correlations were used to investigate rates of stillbirth with maternal co-morbidities (obesity, diabetes and hypertension) and the use of ART.

## Results

### Study selection and characteristics

**Fig 1:**
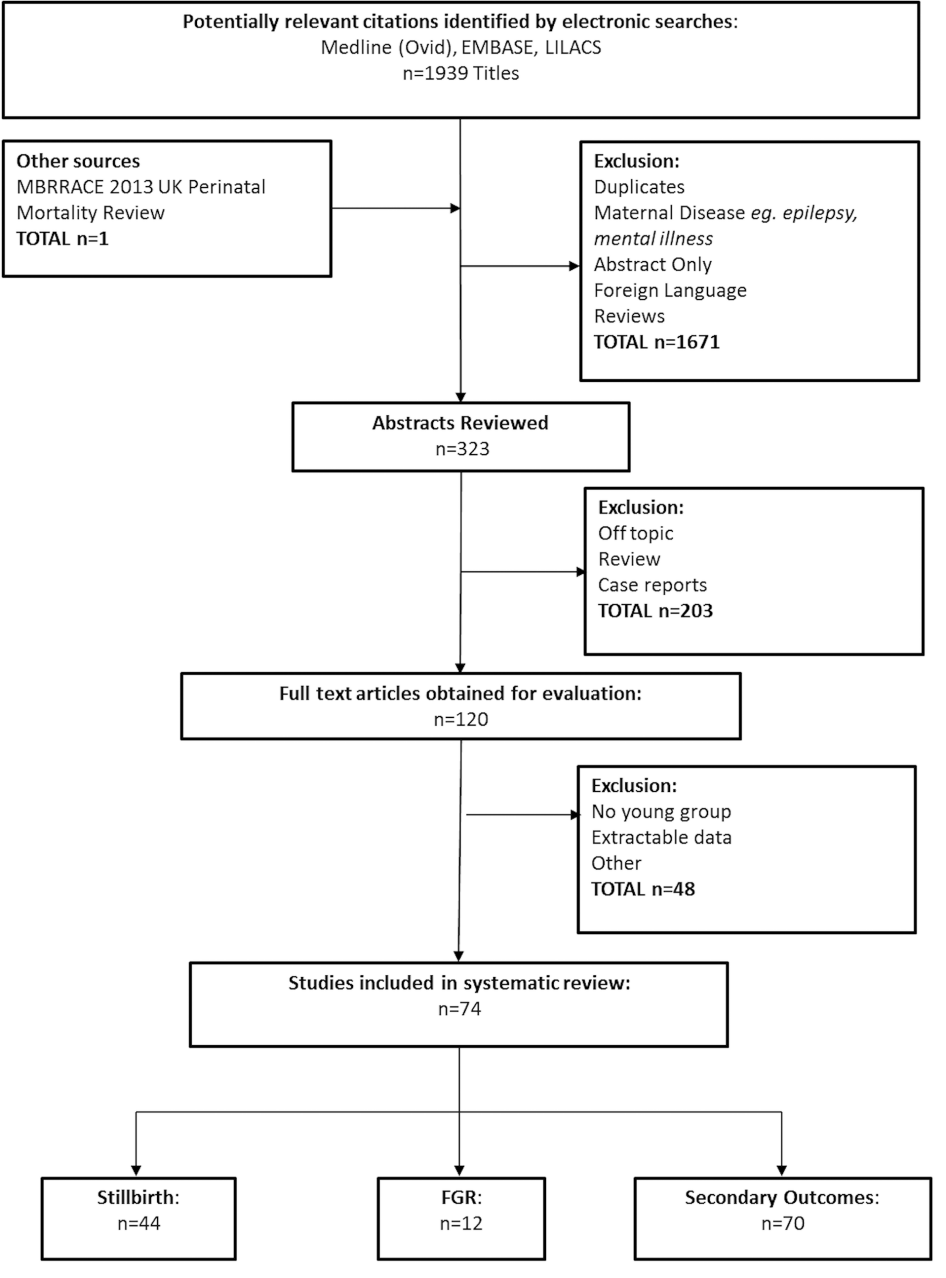
PRISMA flow diagram of systematic review search strategies.

Our systematic search strategy identified 1,940 titles; after removal of duplicates and screening of abstracts 120 full-text articles were fully evaluated. 74 titles related to AMA and pregnancy outcome were included in the final analysis. 44 relating to stillbirth, 12 to FGR and 70 reported on one or more secondary outcomes (Fig 1). The majority of studies (53/74) were conducted in high income countries (HICs) and 62/74 were cohort studies (Summary of study characteristics can be found in S1 Table).

### Risk of bias of included studies

**Fig 2:**
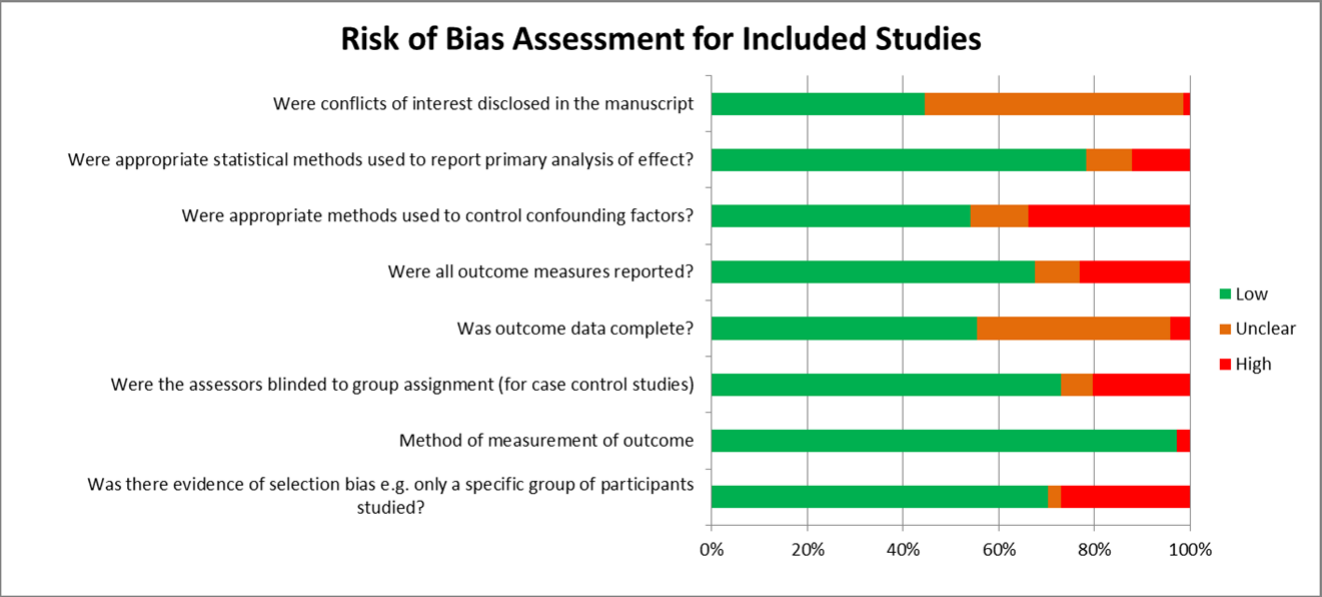
Quality assessment: Risk of bias assessment for included studies in meta-analysis classified as high, low or unclear.

The majority of studies had a low risk of bias in the assessed domains (Fig 2), although it was difficult to assess the completeness of outcome data as the primary and secondary outcomes of interest were often not stated a priori. 68% of studies reported on all outcomes specified in their methods sections. Statistical assessment was considered appropriate for almost 80% of the studies included in this analysis.

### Synthesis of results

**Fig 3:**
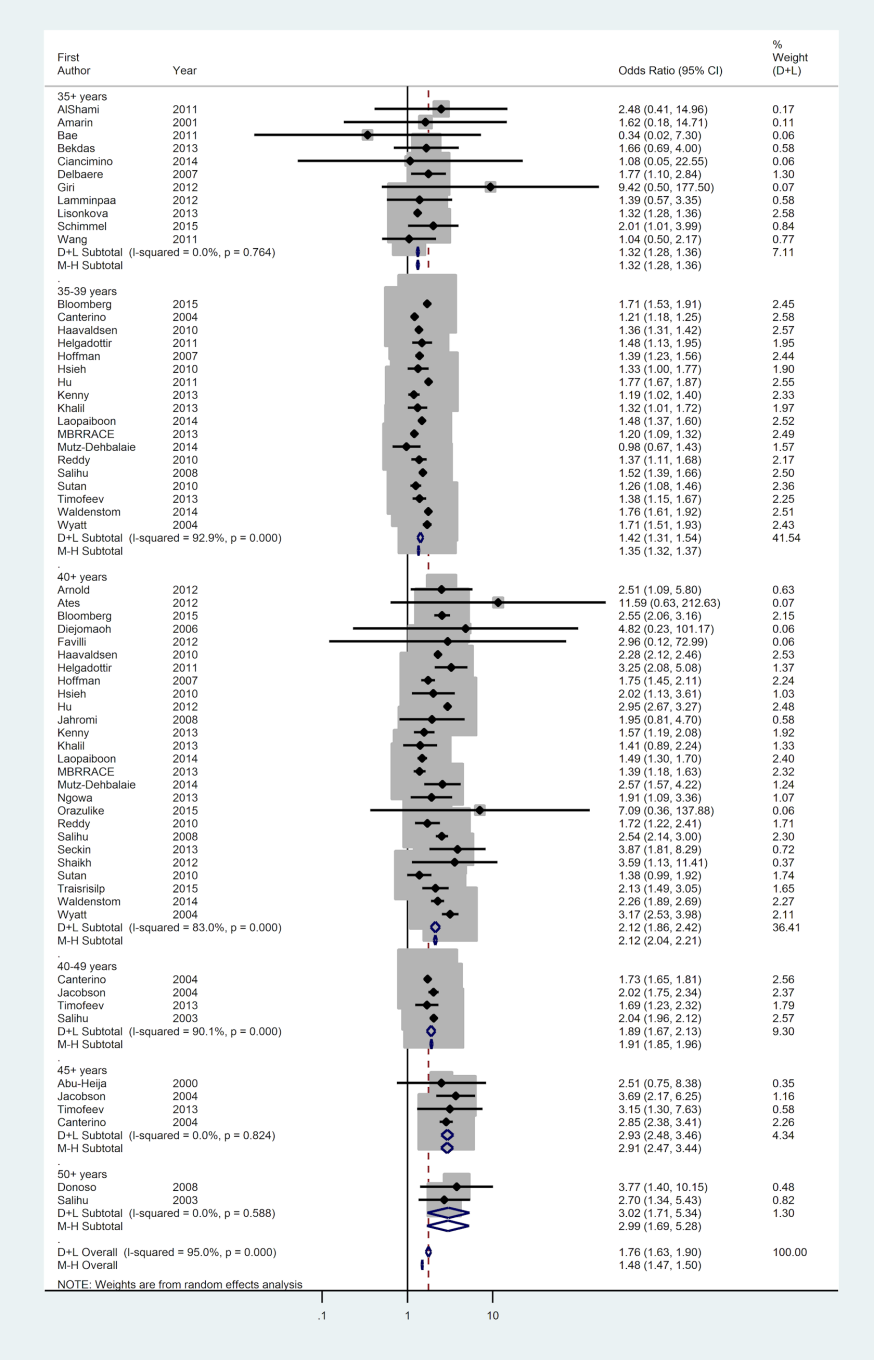
Forest plot of odds ratios of stillbirth stratified by maternal age group (≥35, 35–39, ≥40, 40–49, ≥45 and ≥50 years of age) weighted from random effects analysis shows increased risk of stillbirth in AMA population (OR 1.75; 95%CI 1.62–1.89). Heterogeneity was classified as severe (Cochran’s χ^2^_,_ I^2^ = 95.0%).

44 studies reported the outcome of 44,723,207 births including 185,384 stillbirths giving a stillbirth rate of 0.42%. The AMA population had an increased risk of stillbirth (Odds ratio (OR) 1.75; 95%CI 1.62 to 1.89). There was significant heterogeneity within the data (I^2^ = 95.6%; classification severe). Inspection of the Forest plot against age suggests increasing maternal age increases odds of stillbirth (Fig 3), which was confirmed by meta-regression (R^2^ = 0.61; Fig 3A). Case-control studies gave a slightly higher estimate of the effect of AMA (OR 2.39; 95%CI 1.57 to 3.66) compared to cohort studies (OR 1.73; 95%CI 1.6 to 1.87), with lower heterogeneity (I^2^ = 46.8%; classification moderate; S1 Fig). There were no differences with geographical region (S3 Fig) or year of study. A contour enhanced funnel plot does not support the hypothesis that small study effects are related to the statistical significance of this result (Fig 3B; Harbord’s Test, *p* = 0.56); this observation is supported by the similarity between the output of random and fixed effects meta-analysis.

**Fig 4:**
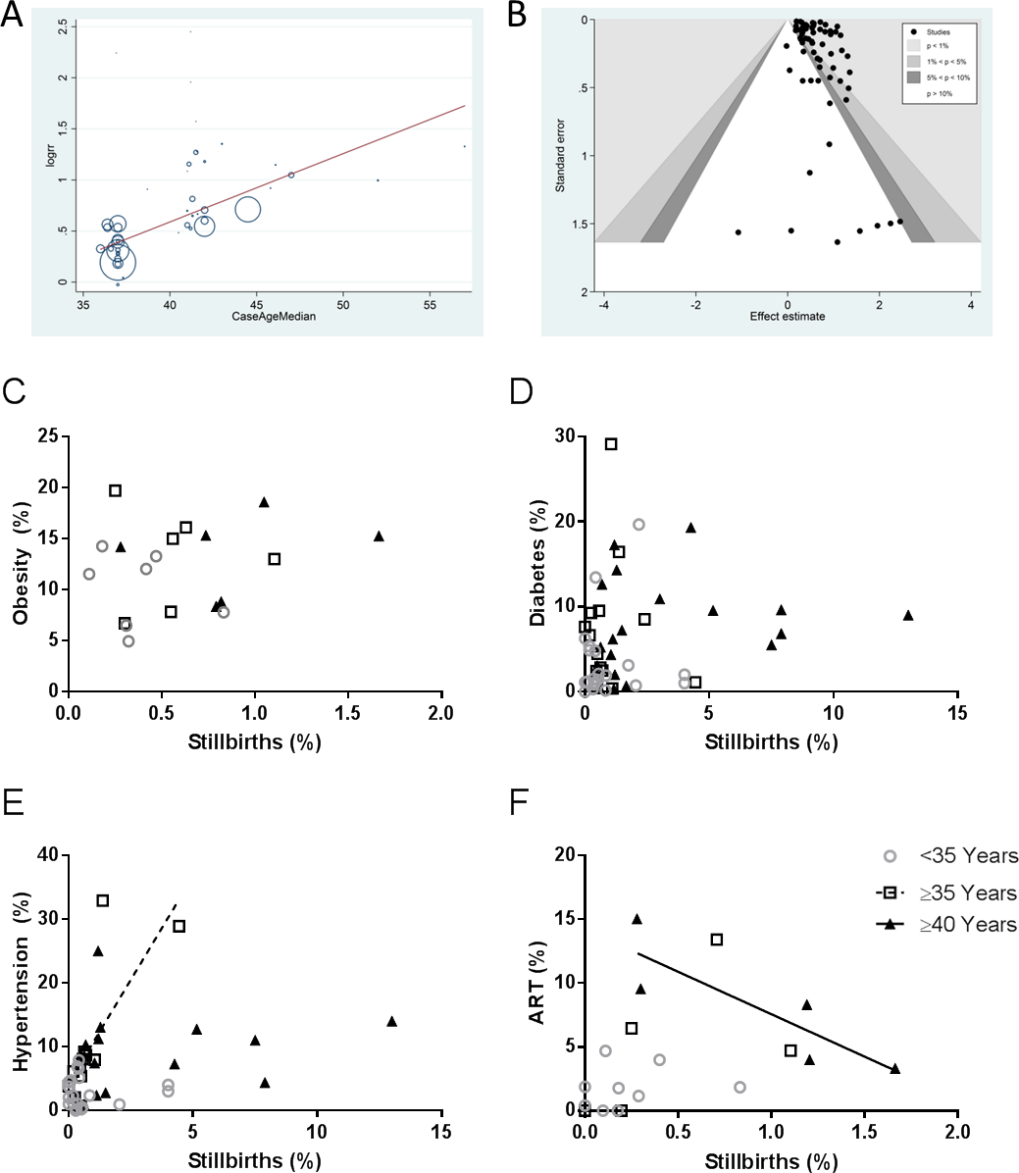
Stillbirth and co-morbidities. A) Meta-regression showed a linear association between stillbirth rates and maternal age (R^2^ = 0.45) and B) contour enhanced funnel plot representing no small study effects (Harbord’s test). Correlation between population rates (% of total population) of maternal co-morbidities C) obesity, D) diabetes, E) hypertension and F) assisted reproductive therapies with stillbirth for mothers ages <35, ≥35 and ≥40 years of age. Population rates of stillbirth were positively correlated with rates of hypertension in ≥35 year old population only (*p* = 0.002) and negatively correlated with ART in the ≥40 year old population only (*p* = 0.017). Spearman rank correlations.

There was no association between the rate of stillbirth in AMA mothers and the prevalence of maternal morbidities (obesity and diabetes) in in the study populations with the exception of hypertension which was positively correlated (Spearman rank, *p* = 0.002) in women aged 35–40 (Fig 4C–4E). The use of ART in mothers ≥40 years was negatively correlated to the rate or stillbirth (Spearman rank, *p* = 0.017) but showed no relationship with stillbirth rate for mothers < or ≤35 years (Fig 4F). The PAR for stillbirth was 4.7% in AMA mothers ≥35 and 2.7% for AMA ≥40 years of age.

12 studies reported 14,019 FGR infants out of 346,189 births (4.0%). Overall, AMA was associated with an increased risk of FGR (OR 1.23; 95%CI 1.01–1.52) although there was significant heterogeneity within the data (I^2^ = 87.5%; classification severe). Inspection of the forest plot against age suggests increasing AMA only significantly increases the risk of FGR over the age of 40 (OR 1.53; 95%CI 1.07 to2.20; S3 Fig). Meta-regression demonstrated a stronger correlation between FGR and AMA than for stillbirth (R^2^ = 0.80%).

A summary of the meta-analysis of secondary outcomes is shown in Table 1. The majority of secondary outcomes: SGA infants, LBW infants, preterm birth, neonatal deaths, NICU admission, preeclampsia and placental abruption and GDM were more frequent in women with AMA. There was no relationship between neonatal acidosis or VLBW infants with AMA, although this may results from fewer studies reporting these outcomes. Only NICU admission and neonatal deaths had a significant correlation to increasing maternal age. All outcomes showed significant heterogeneity (I^2^ ranging from 79.3–100%). For all outcomes, case control studies reduced heterogeneity and gave larger estimates of effect size. There were no differences with geographical location (continent) or year of study on secondary outcomes.

**Table 1:** Meta-analysis of secondary outcomes in AMA.

	Overall Meta-Analysis					Age Group (Years)						
Condition	Number of Studies	Number of Births	Number of cases (%)	I^2^ (%)	Overall OR (95% CI)	≥35	35–39	≥40	40–49	≥45	≥50	Meta-Regression (R^2^)
SGA	25	16,949,750	2,028,565 (12.0)	98.7	**1.16** **(1.06–1.27)**	1.22 (0.86–1.73)	1.06 (0.86–1.31)	1.20 (1.07–1.33)	0.97 (0.96–0.98)	1.57 (1.17–2.10)	NS	0.0007
LBW (<2500g)	35	18,360,387	1,085,677 (5.9)	98.5	**1.37** **(1.26–1.50)**	1.44 (1.09–1.88)	1.10 (0.83–1.46)	1.46 (1.18–1.80)	1.40 (1.38–1.42)	2.05 (1.67–2.52)	1.33 (1.02–1.74)	0.10
VLBW (<1500g)	14	16,826,886	231,534 (1.4)	100	**1.59** **(0.65–3.94)**	1.42 (0.93–2.18)	1.47 (1.21–1.79)	1.81 (1.37–2.39)	3.09 (0.50–18.97)	1.62 (0.97–2.71)	1.07 (0.59–1.94)	*
PTB (<37 wks)	46	24,551,442	2,501,490 (10.2)	98.4	**1.45** **(1.38–1.53)**	1.52 (1.26–1.83)	1.28 (1.17–1.40)	1.52 (1.44–1.61)	1.28 (1.26–1.30)	2.01 (1.50–2.68)	1.17 (0.90–1.54)	0.17
Neonatal Death	27	13,245,799	45,391 (0.3)	82.6	**1.48** **(1.30–1.67)**	2.29 (0.93–5.66)	1.21 (1.04–1.41)	1.41 (1.14–1.75)	1.62 (1.38–1.90)	1.95 (1.24–3.06)	10.26 (5.85–17.97)	0.81
NICU Admission	20	645,661	5,324 (8.3)	90.8	**1.49** **(1.34–1.66)**	1.85 (1.09–3.17)	1.18 (1.11–1.25)	1.38 (1.31–1.47)	NS	1.81 (1.40–2.33)	NS	0.54
Neonatal Acidosis	2	1,014	181 (17.8)	96.3	**1.15** **(0.18–7.33)**	1.15 (0.18–7.33)	NS	NS	NS	NS	NS	*
PE	38	10,230,730	14,019 (3.2)	98.7	**1.99** **(1.65–2.36)**	1.18 (0.86–1.60)	1.63 (1.09–2.44)	2.42 (1.85–3.55)	1.65 (1.09–2.48)	3.67 (1.12–11.97)	2.47 (1.83–3.34)	0.11
Placental Abruption	32	8,843,049	52,624 (0.6)	79.3	**1.52** **(1.35–1.70)**	1.17 (0.73–1.86)	1.38 (1.13–1.69)	2.02 (1.54–2.65)	1.44 (1.09–1.91)	2.29 (0.93–5.65)	1.55 (0.64–3.74)	0.12
GDM	28	1,694,232	38078 (2.2)	96.2	**2.85** **(2.46–3.32)**	1.87 (1.65–2.12)	1.95 (1.27–3.02)	3.76 (2.99–4.73)	3.82 (2.89–5.04)	4.81 (2.65–8.72)	NS	0.44

*Too few studies to perform meta-regression.

NS = No Studies. SGA = Small for Gestational Age; LBW = Low Birth Weight; PTB = Pre-Term Birth; NICU = Neonatal Intensive Care Unit; PE = Preeclampsia; GDM = Gestational Diabetes Mellitus

Data regarding pregnancy primary or secondary outcomes was split between nulliparous and multiparous mothers in only 9 of the included papers. Meta-regression analysing the effect of parity on primary or secondary outcomes was only conducted if data were extractable from ≥5 studies per outcome (Table 2). There was no consistent relationship between parity and the effects of AMA. Rates of stillbirth did not vary with maternal age in nulliparous women, but was more common in AMA multiparous women compared to multiparous controls (OR 1.88; 95%CI 1.54–2.28). Conversely, the incidence of LBW was greater in AMA nulliparous women than nulliparous controls (OR 2.28; 95%CI 1.25–4.13), however this affect was absent in multiparous women. Parity had no apparent effects on outcome rates for PE, PTB or GDM between AMA and control women which were elevated in both nulliparous and multiparous AMA mothers.

**Table 2:** Meta-analysis of parity effects on primary and secondary outcomes.

Outcome	# Studies	Total Births	#Con		#AMA		Primips		Meta-analysis		Multips		Meta-analysis	
			P	M	P	M	Con	AMA	OR (95%CI)	I^2^	Con	AMA	OR (95%CI)	I^2^
**Stillbirth**	5	221,581	46,997	51,933	8,024	22,866	227 (0.48)	33 (0.41)	1.29 (0.64–2.57)	57.1	189 (0.36)	159 (0.70)	1.88 (1.54–2.29)	0.0
**LBW**	7	39,098	24,291	10,129	1,545	3,133	1512 (6.22)	164 (10.61)	2.28 (1.25–4.13)	85.9	639 (6.31)	265 (8.46)	1.16 (0.98–1.37)	0.0
**PE**	7	160,076	60,734	63,194	10,149	25,999	2582 (4.25)	568 (5.60)	1.82 (1.04–2.73)	68.1	927 (1.47)	534 (2.05)	2.11 (1.38–3.23)	77.6
**PTB**	7	39,098	24,291	10,129	1,545	3,133	1184 (7.76)	176 (11.39)	1.68 (1.04–2.73)	82.8	803 (7.93)	356 (11.36)	1.23 (1.03–1.46)	18.2
**GDM**	5	15,725	59,966	61,946	10,082	25,281	2822 (4.71)	692 (6.86)	2.23 (1.32–3.75)	83.9	2822 (4.71)	856 (3.39)	2.90 (1.02–8.22)	98.0

LBW = Low Birth Weight; PE = Preeclampsia; PTB = Pre-Term Birth; GDM = Gestational Diabetes Mellitus

## Discussion

### Main findings

This meta-analysis not only describes an increased risk of stillbirth in AMA, but also suggests a relationship between increasing maternal age and the magnitude of risk of stillbirth. This increased risk cannot be accounted for by increased prevalence of maternal comorbidities and was despite a negative association between rate of stillbirth and use of ART in mothers ≤40 years. The not unsubstantial contribution of AMA to the overall total of stillbirths is estimated at 4.7% (of stillbirths attributable to AMA ≥35 years). Furthermore, maternal age is found to significantly increase the frequency of adverse pregnancy outcomes including FGR, preeclampsia and placental abruption.

Despite the association between AMA and stillbirth, no studies distinguished between different causes of stillbirth, although one study only included unexplained stillbirths [21] the most frequent classification in AMA [22] As only 12% of stillbirths are associated with congenital abnormality, it is unlikely that the increased risk of stillbirth in AMA mothers is attributable to anomalous stillbirths. Therefore, the observation that AMA is also associated with an increased risk of disorders such as placental abruption, preeclampsia and FGR suggests that these conditions related to placental pathology/dysfunction contribute to the elevated stillbirth risk in AMA mothers. This is strengthened by the observation that FGR and preeclampsia also had a similar relationship with increased maternal age to that seen for stillbirth.

### Strengths and limitations

This meta-analysis includes data from a large number of births and is the largest systematic review of the effects of AMA on pregnancy outcome. Furthermore, this study is the first study of AMA to use meta-regression to evaluate maternal age as a continuous variable showing that that elevated risks for these pregnancy outcomes are exacerbated with advancing maternal age. Secondly, we have also been able to extract data to address potential reasons for adverse outcome in AMA pregnancies. By doing so, we have demonstrated that there was no apparent relationship between the incidence of stillbirth and the prevalence of maternal conditions known to be associated with stillbirth such as maternal obesity, diabetes and hypertension in agreement with previous studies [9–11] Furthermore, the increased risk of stillbirth in the AMA population is despite a negative correlation with use of ART. Although IVF is typically associated with increased stillbirth risk [23] this does not appear to be related to the increased risk seen in AMA in these studies. This may be due to rigorous clinical criteria for eligibility for ART in women of AMA or the use of donor egg/ donor sperm which was not specifically reported in these studies. Alternatively, women who use ART may be regarded as high-risk pregnancies and have additional medication or clinical care which reduce the risk of stillbirth. Taken together, these findings highlight maternal age or ageing to be an independent factor associated with adverse pregnancy outcome.

This meta-analysis identified significant heterogeneity between different studies that has previously led authors of systematic reviews on the topic to avoid drawing summary estimates of the effect of AMA. In part, the large I^2^ values obtained reflect the very large number of women included in the meta-analysis, which has led other meta-analyses to report the τ^2^ value–a more conservative measure of heterogeneity [24] We elected to use the I^2^ value as the magnitude of changes has been classified previously and it is more easily comparable to published studies and the magnitude is comparable to other systematic reviews and meta-analyses of stillbirth risk (72–99%) [25,26]. Case-control studies, which can control for confounding factors more effectively, had lower levels of heterogeneity than cohort studies, suggesting that study design may have played a role in the heterogeneity of estimated risk for AMA mothers.

The high heterogeneity may come from a variety of sources. Different studies applied different definitions of stillbirth, ranging from 16 to 24 weeks gestation, with several studies not disclosing their classification which could have impacted study heterogeneity [27] Differences were not seen in geographically suggesting that including degree of economic development, inter-study sample sizes and local geographical factors did not significantly affect rates of stillbirth in AMA women. Studies used for analysis of secondary outcomes similarly showed significant variation in the definitions employed. Where the definition was included in the manuscript, we were able to control for this, but in the absence of a definition we had to use the authors’ classification.

Similarly, it was noted that there were fewer studies available from low and middle income countries (LMICs) which have higher rates of stillbirth and adverse pregnancy outcomes than HICs. Nevertheless, the recent Lancet Ending Preventable Stillbirth Series found that AMA has a significant contribution to stillbirth LMICs, particularly in sub-Saharan Africa and South Asia [28] This reflects a paucity of research into the causes of stillbirth in LMICs, which currently focus on other factors that have larger impacts on obstetric outcome, such as access to basic maternity care and management of birth. Further studies are required to confirm the relationship between AMA and increased risk of stillbirth in LMICs, and how AMA interacts with other risk factors for stillbirth in this setting.

Although we only included data that distinguished between mothers less than or equal/over 35 years of age, different studies used different definitions of AMA and had varying degrees of resolution. Where possible, we extracted data within the specified age groups 35–39, 40–44 and ≥45 years—although larger age groups were often used restricting this classification. Furthermore, the age of control populations also varied. Where possible, we combined reported data for maternal ages 20–30 years, although some studies only reported mothers <35 years combined, with or without exclusion of mothers <20 years or used a specific age range that showed the lowest rates of their observed outcome in their population (often 24–27 years).

Included studies also exerted varying degrees of control for confounding factors on stillbirth rates. Some studies excluded multiple gestations, fetal anomalies and pre-existing maternal medical conditions, whereas others did not have access to this level of data. Use of ART also varied between studies. We excluded studies whose focus was purely on outcomes of ART pregnancies but there was variation on whether ART pregnancies were included or specifically excluded within each study.

### Interpretation

The results from this extensive data set suggest that little will be gained from further retrospective epidemiological studies associating adverse outcome with AMA. The relationship between AMA and increased rate of stillbirth has been established and is consistently observed in different geographical regions, irrespective of the methodology employed. Studies are required to identify the impact of co-morbidities and the relationship between sociodemographic factors and the increased risk of stillbirth in AMA mothers. Furthermore, descriptive and mechanistic studies are required to determine the causes of stillbirths and the underlying pathological processes that increase fetal morbidity and mortality in AMA mothers. The association of maternal age with disorders that are strongly related to stillbirth and placental dysfunction (FGR, PE and placental abruption) makes this a logical avenue to explore. The absence of routinely collected data on the cause of stillbirths using a modern classification system means that this would be challenging using population level data. One retrospective cohort study of 15,402 women attempted to explore this link and demonstrated that two markers of utero-placental insufficiency were significantly higher in AMA mothers (fetal distress and caesarean section for fetal distress) [29] Despite this observation, the authors concluded that increased stillbirth rates in AMA were not explained by fetal distress secondary to utero-placental insufficiency and all other markers measured were not different including birthweight <10^th^ centile and 5 minute Apgar <7. This may be in part due to relying only on variable antepartum testing methods and results from historical data which are only partially reflective of placental insufficiency.

The effect of parity on rates of pregnancy outcomes in AMA is an important avenue to explore further. Existing literature is limited and therefore the small number of studies included in these analysis means that limited conclusions can be drawn. However, we did not see a consistent relationship between parity and adverse perinatal outcome. Thus, the findings of increased perinatal mortality in AMA mothers cannot be restricted to nulliparous or multiparous women.

The possibility that AMA is linked to stillbirth by another confounding factor must be considered. One such variable is advanced paternal age, which has received considerably less attention, but is commonly coupled with AMA. A study by Alio et al. found a 24% increase in rate of stillbirth with paternal age between 40 and 45 years old, and a 50% increase with paternal age ≥45 years, independent of maternal age [30] Further studies are needed which incorporate paternal age as a co-variate to determine its contribution to the increased risk of stillbirth in AMA mothers.

Although placental dysfunction has a key role in the aetiology of stillbirth, preeclampsia and FGR, it is complicated and incompletely understood. Furthermore, comparatively little is known about the relationship between AMA and placental function. Our recent studies of AMA pregnancies identified signs of accelerated placental ageing, altered nutrient transport and vascular function compared to a control group [31] Similar features were also detected in a mouse model of AMA, which has a high rate of FGR and late fetal death. This observation agrees with the most commonly suggested explanation of adverse outcome in AMA pregnancy which relates to maternal vascular dysfunction, supported by evidence in a rat model of AMA [32] Another theory is the reduced genetic quality of the ageing oocyte [33] The relationship between oocyte age and pregnancy outcome is difficult to investigate, as oocyte donation (with younger eggs) is an independent factor for adverse outcome [34] However, animal models have found relationships between oocyte ageing and early placental development which may lead to altered placental function [35] Both theories provide a plausible link between AMA and adverse outcomes mediated by abnormal placental development, structure and function. The strong association between AMA and GDM is likely to be independent of placental function and therefore have an independent aetiology which should also be investigated further but may be related to increased BMI [36] or use of assisted reproductive therapies in AMA pregnancies [37]

## Conclusion

To the best of our knowledge, this is the largest and most comprehensive systematic review investigating AMA and pregnancy outcome, and the only one that has focused on potential factors and co-pathologies underlying the increased risk of stillbirth observed in AMA pregnancies. We have shown that AMA has an association with rates of stillbirth and FGR. Despite the large degree of heterogeneity, the extensive meta-analysis has consistent themes that we believe to be true effects of increasing maternal age on stillbirth rates and associated pregnancy complications. Most of the conditions related to AMA identified in this analysis have strong biological associations with placental dysfunction providing a logical avenue for future study. Further understanding the mechanisms underpinning this increased risk of stillbirth in AMA may provide novel tools to identify women at the highest risk of stillbirth, so that intervention may be applied to improve outcomes for this high risk population. Our data also suggest that women over the age of 40 are at greater risk of stillbirth than women from 35–39, implying that any intervention might initially be targeted towards that age group.

## Supporting information

S1 Table Systematic review articles. Systematic Review Articles.
(DOCX)

S1 Fig Forest plot of odds ratios of stillbirth stratified by study type (case control or cohort) weighted from random effects analysis shows increased effect of AMA on stillbirth rates in case control (OR 2.39; 95%CI 1.57–3.66) than in cohort studies (OR 1.73; 95%CI 1.60–1.87). Heterogeneity was classified as severe for cohort studies (I^2^ = 95.6%) and moderate for case control studies (I^2^ = 48.0%). Overall heterogeneity was classified as severe (Cochran’s χ^2^_,_ I^2^ = 95.0%).
(TIF)

S2 Fig Forest plot of odds ratios of stillbirth stratified by geographical location (Middle East, Far East, Europe, Asia, North America, East Asia, Multi-country, South America, Australasia and Africa) weighted from random effects analysis shows no differences on effects of AMA on stillbirth rates. Heterogeneity was classified as severe for (Cochran’s χ^2^_,_ I^2^ = 95.0%).
(TIF)

S3 Fig Forest plot of odds ratios of fetal growth restriction (FGR) stratified by maternal age group (≥35, 35–39, ≥40, 40–49, ≥45 and ≥50 years of age) weighted from random effects analysis shows increased risk of FGR in mothers ≥40 years of age (OR 1.53; 95%CI 1.07–2.02). Heterogeneity was classified as severe (Cochran’s χ^2^_,_ I^2^ = 87.5%).
(TIF)

S1 Appendix Search strategy for Ovid. (DOCX)

S2 Appendix Systematic review and meta-analysis protocol. (DOCX)

S3 Appendix PRISMA checklist. (DOC)

## References

[1] RCOG (2011) Statement on later maternal age. In: RCOG, editor. Compaining and Opinions.

[2] ONS (2014) Birth Summary Tables, England and Wales, 2013. Office for National Statistics.

[3] Guedes M, Canavarro MC (2014) Characteristics of primiparous women of advanced age and their partners: a homogenous or heterogenous group? Birth 41: 46–55. doi: 10.1111/birt.12089 24654637 10.1111/birt.12089

[4] Cleary-Goldman J, Malone FD, Vidaver J, Ball RH, Nyberg DA, et al (2005) Impact of maternal age on obstetric outcome. Obstet Gynecol 105: 983–990. doi: 10.1097/01.AOG.0000158118.75532.51 15863534 10.1097/01.AOG.0000158118.75532.51

[5] Kenny LC, Lavender T, McNamee R, O'Neill SM, Mills T, et al (2013) Advanced maternal age and adverse pregnancy outcome: evidence from a large contemporary cohort. Plos One 8: e56583 doi: 10.1371/journal.pone.0056583 23437176 10.1371/journal.pone.0056583 PMC3577849

[6] Khalil A, Syngelaki A, Maiz N, Zinevich Y, Nicolaides KH (2013) Maternal age and adverse pregnancy outcome: a cohort study. Ultrasound in Obstetrics & Gynecology 42: 634–643. 24273200 10.1002/uog.13234

[7] Salihu HM, Wilson RE, Alio AP, Kirby RS (2008) Advanced maternal age and risk of antepartum and intrapartum stillbirth. Journal of Obstetrics and Gynaecology Research 34: 843–850. doi: 10.1111/j.1447-0756.2008.00855.x 18834344 10.1111/j.1447-0756.2008.00855.x

[8] Giri A, Srivastav VR, Suwal A, Tuladhar AS (2012) Advanced maternal age and obstetric outcome. Nepal Medical College Journal: NMCJ 15: 87–90. 24696922

[9] Bahtiyar M, Funai E, Norwitz E, Buhimschi C, Rosenberg V, et al (2006) Advanced maternal age (AMA) is an independent predictor of intrauterine fetal death at term. Am J Obstet Gynecol 195: S209–S209.

[10] Odibo AO, Nelson D, Stamilio DM, Sehdev HM, Macones GA (2006) Advanced maternal age is an independent risk factor for intrauterine growth restriction. Am J Perinatol 23: 325–328. doi: 10.1055/s-2006-947164 16799913 10.1055/s-2006-947164

[11] Lamminpaa R, Vehvilainen-Julkunen K, Gissler M, Heinonen S (2012) Preeclampsia complicated by advanced maternal age: a registry-based study on primiparous women in Finland 1997–2008. BMC Pregnancy & Childbirth 12: 47. 22687260 10.1186/1471-2393-12-47 PMC3495042

[12] Bayrampour H, Heaman M (2010) Advanced maternal age and the risk of cesarean birth: a systematic review. Birth 37: 219–226. doi: 10.1111/j.1523-536X.2010.00409.x 20887538 10.1111/j.1523-536X.2010.00409.x

[13] Huang L, Sauve R, Birkett N, Fergusson D, van Walraven C (2008) Maternal age and risk of stillbirth: a systematic review. Canadian Medical Association Journal 178: 165–172. doi: 10.1503/cmaj.070150 18195290 10.1503/cmaj.070150 PMC2175002

[14] Carolan M (2013) Maternal age > = 45 years and maternal and perinatal outcomes: A review of the evidence. Midwifery 29: 479–489. doi: 10.1016/j.midw.2012.04.001 23159159 10.1016/j.midw.2012.04.001

[15] Flenady V, Middleton P, Smith GC, Duke W, Erwich JJ, et al (2011) Stillbirths: the way forward in high-income countries. Lancet 377: 1703–1717. doi: 10.1016/S0140-6736(11)60064-0 21496907 10.1016/S0140-6736(11)60064-0

[16] Stroup DF, Berlin JA, Morton SC, Olkin I, Williamson GD, et al (2000) Meta-analysis of observational studies in epidemiology—A proposal for reporting. Jama-Journal of the American Medical Association 283: 2008–2012. 10.1001/jama.283.15.2008 10789670

[17] Unterscheider J, Daly S, Geary MP, Kennelly MM, McAuliffe FM, et al (2014) Definition and management of fetal growth restriction: a survey of contemporary attitudes. European Journal of Obstetrics & Gynecology and Reproductive Biology 174: 41–45. 24360357 10.1016/j.ejogrb.2013.11.022

[18] Sanderson S, Tatt LD, Higgins JPT (2007) Tools for assessing quality and susceptibility to bias in observational studies in epidemiology: a systematic review and annotated bibliography. International Journal of Epidemiology 36: 666–676. doi: 10.1093/ije/dym018 17470488 10.1093/ije/dym018

[19] Harris RJ, Deeks JJ, Altman DG, Bradburn MJ, Harbord R, et al (2008) Metan- fixed- and random-effects meta analysis. The Stata Journal 8: 3–28.

[20] Higgins JPT, Thompson SG, Deeks JJ, Altman DG (2003) Measuring inconsistency in meta-analyses. British Medical Journal 327: 557–560. doi: 10.1136/bmj.327.7414.557 12958120 10.1136/bmj.327.7414.557 PMC192859

[21] Sutan R, Campbell D, Prescott GJ, Smith WC (2010) The risk factors for unexplained antepartum stillbirths in Scotland, 1994 to 2003. J Perinatol 30: 311–318. doi: 10.1038/jp.2009.158 19829298 10.1038/jp.2009.158 PMC2864419

[22] Bahtiyar MO, Funai EF, Rosenberg V, Norwitz E, Lipkind H, et al (2008) Stillbirth at term in women of advanced maternal age in the United States: when could the antenatal testing be initiated? Am J Perinatol 25: 301–304. doi: 10.1055/s-2008-1076605 18437644 10.1055/s-2008-1076605

[23] Wisborg K, Ingerslev HJ, Henriksen TB (2010) IVF and stillbirth: a prospective follow-up study. Hum Reprod 25: 1312–1316. doi: 10.1093/humrep/deq023 20179321 10.1093/humrep/deq023

[24] Oteng-Ntim E, Meeks D, Seed PT, Webster L, Howard J, et al (2015) Adverse maternal and perinatal outcomes in pregnant women with sickle cell disease: systematic review and meta-analysis. Blood 125: 3316–3325. doi: 10.1182/blood-2014-11-607317 25800049 10.1182/blood-2014-11-607317

[25] Berhan Y, Berhan A (2014) A meta-analysis of selected maternal and fetal factors for perinatal mortality. Ethiop J Health Sci 24 Suppl: 55–68. 25489183 10.4314/ejhs.v24i0.6s PMC4249209

[26] Lamont K, Scott NW, Jones GT, Bhattacharya S (2015) Risk of recurrent stillbirth: systematic review and meta-analysis. Bmj-British Medical Journal 350. 10.1136/bmj.h3080 26109551

[27] Cartlidge PH, Stewart JH (1995) Effect of changing the stillbirth definition on evaluation of perinatal mortality rates. Lancet 346: 486–488. 7637485 10.1016/s0140-6736(95)91327-0

[28] Lawn JE, Blencowe H, Waiswa P, Amouzou A, Mathers C, et al (2016) Stillbirths: rates, risk factors, and acceleration towards 2030. Lancet 387: 587–603. doi: 10.1016/S0140-6736(15)00837-5 26794078 10.1016/S0140-6736(15)00837-5

[29] Miller DA, Robertson P, Smith W, Schwartz M, Parer J, et al (2005) Is advanced maternal age an independent risk factor for uteroplacental insufficiency? American Journal of Obstetrics and Gynecology 192: 1974–1982. doi: 10.1016/j.ajog.2005.02.050 15970865 10.1016/j.ajog.2005.02.050

[30] Alio AP, Salihu HM, McIntosh C, August EM, Weldeselasse H, et al (2012) The effect of paternal age on fetal birth outcomes. Am J Mens Health 6: 427–435. doi: 10.1177/1557988312440718 22564913 10.1177/1557988312440718

[31] Lean SC, Heazell AEP, Dilworth MR, Mills TA, Jones RL (2017) Placental Dysfunction Underlies Increased Risk of Fetal Growth Restriction and Stillbirth in Advanced Maternal Age Women. Scientific Reports Epub Ahead of Print. 10.1038/s41598-017-09814-w PMC5574918 28852057

[32] Care AS, Bourque SL, Morton JS, Hjartarson EP, Davidge ST (2015) Effect of advanced maternal age on pregnancy outcomes and vascular function in the rat. Hypertension 65: 1324–1330. doi: 10.1161/HYPERTENSIONAHA.115.05167 25916720 10.1161/HYPERTENSIONAHA.115.05167

[33] Hunt PA, Hassold TJ (2008) Human female meiosis: what makes a good egg go bad? Trends Genet 24: 86–93. doi: 10.1016/j.tig.2007.11.010 18192063 10.1016/j.tig.2007.11.010

[34] Younis JS, Laufer N (2015) Oocyte Donation Is an Independent Risk Factor for Pregnancy Complications: The Implications for Women of Advanced Age. Journal of Womens Health 24: 127–130. 10.1089/jwh.2014.4999 25646636

[35] Lopes FL, Fortier AL, Darricarrere N, Chan D, Arnold DR, et al (2009) Reproductive and epigenetic outcomes associated with aging mouse oocytes. Hum Mol Genet 18: 2032–2044. doi: 10.1093/hmg/ddp127 19293340 10.1093/hmg/ddp127

[36] Makgoba M, Nelson SM, Savvidou M, Messow CM, Nicolaides K, et al (2011) First-Trimester Circulating 25-Hydroxyvitamin D Levels and Development of Gestational Diabetes Mellitus. Diabetes Care 34: 1091–1093. doi: 10.2337/dc10-2264 21454797 10.2337/dc10-2264 PMC3114479

[37] Ashrafi M, Gosili R, Hosseini R, Arabipoor A, Ahmadi J, et al (2014) Risk of gestational diabetes mellitus in patients undergoing assisted reproductive techniques. European Journal of Obstetrics & Gynecology and Reproductive Biology 176: 149–152. 24630294 10.1016/j.ejogrb.2014.02.009

