# Supplementary material for: Advanced maternal age and adverse pregnancy outcomes: A systematic review and meta-analysis
Source: PLoS One. 2017 Oct 17;12(10):e0186287. doi: 10.1371/journal.pone.0186287 (PMC5645107; doi:10.1371/journal.pone.0186287)
Supplement: S1 Table — Systematic Review Articles. (DOCX) [file pone.0186287.s001.docx]

**Table S1 - Systematic Review Articles**

| Author | Date | Study Type | Place | Total Births | Control | AMA | Primary Outcomes | Secondary Outcomes |
| --- | --- | --- | --- | --- | --- | --- | --- | --- |
| Abu-Heija [38] | 2000 | Case Control | Middle East | 248 | 125 | 123 | SB, FGR | PTB, PE, NND, PND, Plac Abrup, GDM |
| AlShami [39] | 2011 | Cohort | Middle East | 893 | 702 | 191 | SB | PTB, NICU, Plac Abrup, GDM |
| Amarin [40] | 2001 | Cohort | Middle East | 549 | 475 | 74 | SB | LBW, Plac Abrup |
| Arnold [41] | 2012 | Cohort | Australia | 62424 | 60270 | 2154 | SB | SGA |
| Ates [42] | 2013 | Case Control | Europe | 199 | 97 | 102 | SB | SGA, PTB, PE, NICU, Plac Abrup |
| Bae [43] | 2011 | Cohort | Far East | 99 | 64 | 35 | SB |  |
| Başer [44] | 2013 | Cohort | Europe | 818 | 613 | 205 | SB, FGR | LBW, PTB, PE, NICU, NND, PND, Plac Abrup |
| Bayrampur [45] | 2011 | Cohort | Canada | 1780 | 1456 | 324 |  | LBW, SGA, PTB |
| Bekdas [46] | 2013 | Cohort | Europe | 4943 | 4423 | 520 | SB | NND, PND |
| Benli [47] | 2015 | Cohort | Europe | 465 | 309 | 156 | FGR | PTB, PE, PND, GDM |
| Biro [48] | 2012 | Cohort | Australia | 135613 | 101338 | 34275 |  | PE, Plac Abrup, GDM |
| Blomberg [49] | 2015 | Cohort | Europe | 769132 | 694901 | 74231 | SB | SGA, PTB, PE, Plac Abrup |
| Canterino [50] | 2004 | Cohort | USA | 18931897 | 16453715 | 2478182 | SB |  |
| Canto [51] | 2012 | Cohort | Europe | 728 | 370 | 358 |  | LBW, VLBW, SGA, PTB, PE, NICU, PND, GDM |
| Carolan [14] | 2013 | Cohort | Australia | 52197 | 51951 | 246 |  | LBW, SGA, PTB, PE, PND, GDM |
| Chan [52] | 2008 | Cohort | East Asia | 16907 | 16259 | 648 |  | SGA, PTB, PE, LBW, PND, GDM |
| Ciancimino [53] | 2014 | Cohort | Europe | 1349 | 1139 | 210 | SB | LBW, VLBW, PTB, PE, NICU, Plac Abrup, GDM |
| Cleary-Goldman [4] | 2005 | Cohort | USA | 37956 | 29875 | 8081 |  | LBW, PTB, PE, PND, Plac Abrup, GDM |
| De Stefano [54] | 2013 | Cohort | Europe | 930 | 564 | 366 | FGR | LBW, PTB, PE, GDM |
| De Weger [55] | 2011 | Cohort | Europe | 259947 | 235943 | 24004 |  | LBW, SGA, PTB |
| Delbaere [56] | 2007 | Cohort | Europe | 27008 | 24017 | 2991 | SB | LBW, VLBW, SGA, PTB, NND |
| Diejomaoh [57] | 2006 | Cohort | Middle East | 330 | 160 | 170 | SB, FGR | LBW |
| Donoso [58] | 2008 | Cohort | South America | 2831915 | 2831694 | 221 | SB | LBW, VLBW, NND, PND |
| Favilli [59] | 2012 | Cohort | Europe | 630 | 312 | 318 | SB | PTB, NICU |
| Figueredo [60] | 2014 | Cohort | South America | 4753 | 4405 | 348 | FGR | PTB |
| Giri [8] | 2012 | Case Control | Nepal | 184 | 90 | 94 | SB | LBW, PE, NND, PND |
| Gravena [61] | 2012 | Cohort | South America | 1316 | 1167 | 149 |  | LBW |
| Haavaldsen [62] | 2010 | Cohort | Europe | 2070068 | 1844699 | 225369 | SB |  |
| Helgadottir [63] | 2011 | Case Control | Europe | 88147 | 75217 | 12930 | SB |  |
| Hoffman [64] | 2007 | Cohort | USA | 128750 | 110442 | 18308 | SB | PE, GDM |
| Hsieh [65] | 2010 | Cohort | East Asia | 40114 | 34163 | 5951 | SB | LBW, VLBW, SGA, PTB, PE, NICU, NND, PND, Plac Abrup, GDM |
| Hu [66] | 2011 | Cohort | Far East | 932165 | 807039 | 125126 | SB |  |
| Jacobson [67] | 2004 | Cohort | Europe | 912230 | 879146 | 33084 | SB | SGA, PTB, PE, NND, PND, Plac Previa, GDM |
| Jahromi [68] | 2008 | Case Control | Middle East | 423 | 208 | 215 | SB | PTB, PE, Plac Abrup |
| Karabulut [69] | 2013 | Cohort | Europe | 6001 | 5314 | 687 |  | LBW, PTB |
| Kenny [5] | 2013 | Cohort | Europe | 226819 | 185545 | 41274 | SB, FGR | SGA, PTB, NND, PND |
| Khalil [6] | 2013 | Cohort | Europe | 76448 | 55967 | 20481 | SB | SGA, PTB, PE, GDM |
| Klemetti [70] | 2013 | Cohort | Finland | 24861 | 22194 | 2667 |  | LBW, VLBW, PTB, NICU, PND |
| Kozinsky [71] | 2002 | Case Control | Europe | 427 | 217 | 210 | FGR | PTB, NICU, NNA, Plac Abrup, GDM |
| Lamminpaa [11] | 2012 | Cohort | Europe | 17858 | 15465 | 2393 | SB | SGA, PTB, NICU |
| Laopaiboon [72] | 2014 | Cohort | Multi-country | 281773 | 242951 | 38822 | SB | LBW, PTB, NICU, NND, PND |
| Lisonkova [73] | 2013 | Cohort | USA | 6868923 | 5472905 | 1396018 | SB | LBW, VLBW, PTB, NND, PND |
| Ludford [74] | 2012 | Cohort | Australia | 35610 | 26904 | 8706 | FGR | SGA, PTB, PND, Plac Abrup, GDM |
| Maisonneuve [75] | 2011 | Case Control | Europe | 566 | 261 | 305 |  | NNA |
| MBRACE [76] | 2013 | Cohort | Europe | 691126 | 545885 | 145241 | SB | NND, PND |
| Mehta [77] | 2014 | Case Control | Europe | 139 | 67 | 72 |  | NICU |
| Mutz-Dehbalaie [78] | 2014 | Cohort | Europe | 56703 | 43448 | 13255 | SB | LBW, VLBW, SGA, NND |
| Najomi [79] | 2009 | Cohort | Middle East | 671 | 357 | 314 |  | LBW, SGA, PTB, NICU, PND |
| Ngowa [80] | 2013 | Cohort | Africa | 2454 | 1849 | 605 | SB | LBW, PTB, PE |
| Oboro [81] | 2006 | Case Control | Africa | 463 | 234 | 229 |  | LBW, SGA, PTB, PE, PND, Plac Abrup, GDM |
| Olusanya [82] | 2012 | Case Control | Africa | 2469 | 1860 | 609 |  | PTB, NICU |
| Orazulike [83] | 2015 | Case Control | Africa | 501 | 249 | 252 | SB | LBW, SGA, GDM |
| Panagopoulos [84] | 2006 | Cohort | Europe | 1982 | 1875 | 107 |  | LBW, NICU, PND, GDM |
| Pasupathy [85] | 2010 | Cohort | Europe | 1886 | 1786 | 100 |  | PND |
| Pawde [86] | 2015 | Cohort | India | 753 | 686 | 67 |  | PTB, Plac Abrup |
| Reddy [87] | 2010 | Cohort | USA | 160227 | 132515 | 27712 | SB |  |
| Salihu [88] | 2003 | Cohort | USA | 6553806 | 6284591 | 269215 | SB | LBW, VLBW, SGA, PTB, PE, Plac Abrup |
| Salihu [7] | 2008 | Cohort | USA | 1240712 | 1141259 | 99453 | SB | PE, Plac Abrup |
| Salihu [89] | 2005 | Cohort | USA | 6092954 | 5883766 | 209188 |  | SGA, PTB |
| Schimmel [90] | 2015 | Cohort | Middle East | 24614 | 11349 | 11265 | SB | LBW, SGA, PTB |
| Shaikh [91] | 2012 | Case Control | Middle East | 217 | 104 | 113 | SB | LBW, PTB, NND, PND, Plac Abrup, GDM |
| Sheiner [92] | 2003 | Cohort | Middle East | 71494 | 62059 | 9435 |  | Plac Abruption |
| Shrim [93] | 2010 | Case Control | Canada | 21052 | 19915 | 1137 |  | VLBW, SGA, PTB, NICU |
| Sutan [21] | 2010 | Cohort | Europe | 498154 | 422056 | 76098 | SB |  |
| Tomofeev [94] | 2013 | Cohort | South America | 184691 | 153834 | 30857 | SB | LBW, VLBW, PTB, PE, NICU, NND, PND, Plac Abrup, GDM |
| Traisrisilp [95] | 2015 | Cohort | Far East | 20472 | 19570 | 902 | SB, FGR | LBW, PTB, Plac Previa, GDM |
| Waldenstom [96] | 2014 | Cohort | Europe | 959673 | 848729 | 110944 | SB | SGA, PTB, NND, PND |
| Wang [97] | 2011 | Cohort | Europe | 6651 | 4427 | 2224 | SB | LBW, PTB, PE, Plac Abrup, GDM |
| Wyatt [98] | 2004 | Cohort | Canada | 232254 | 187394 | 44860 | SB |  |
| Yaniv [99] | 2011 | Cohort | Middle East | 50198 | 48759 | 1439 | FGR | LBW, VLBW, PTB, PE, PND, GDM |
| Yogev [100] | 2010 | Cohort | Middle East | 4125 | 1924 | 2201 |  | LBW, VLBW, SGA, PTB, PE, NND, Plac Abrup, NICU, GDM |
| Yuan [101] | 2000 | Cohort | Europe | 15415 | 14816 | 599 |  | LBW, VLBW, PTB, NICU |

*SB = Stillbirth, FGR = fetal growth restriction, SGA = small for gestational age, LBW = low birth weight, VLBW = very low birth weight, PTB = preterm birth, NND = neonatal death, PND = perinatal death, NNA = neonatal acidosis, NICU = neonatal intensive care unit admission; Plac Abrup = Placental abruption.*
