# Supplementary figures and images for: Advanced maternal age and adverse pregnancy outcomes: A systematic review and meta-analysis
Source: PLoS One. 2017 Oct 17;12(10):e0186287. doi: 10.1371/journal.pone.0186287 (PMC5645107; doi:10.1371/journal.pone.0186287)

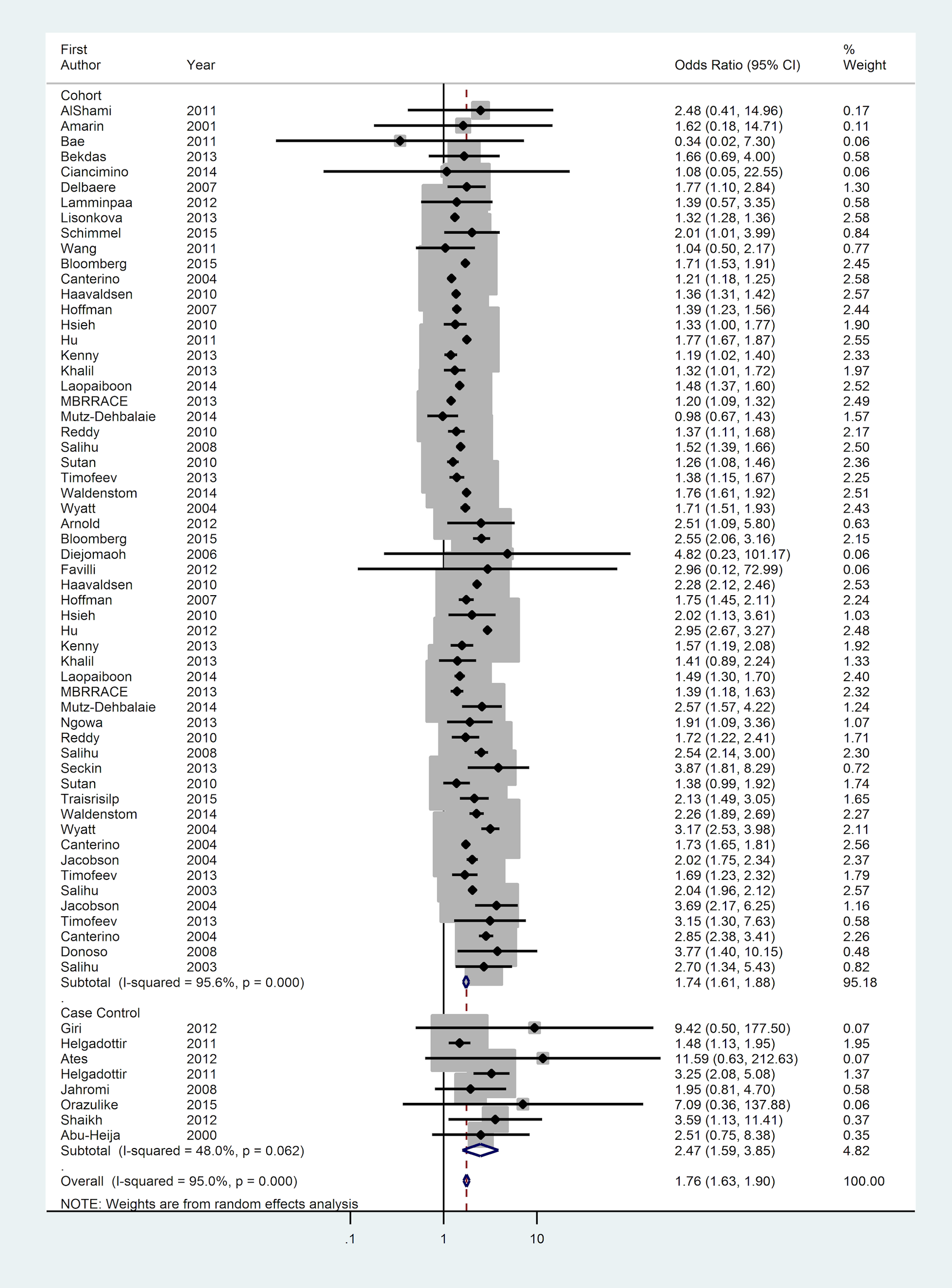

Supplement: S1 Fig — Heterogeneity was classified as severe for cohort studies (I2 = 95.6%) and moderate for case control studies (I2 = 48.0%). Overall heterogeneity was classified as severe (Cochran’s χ2, I2 = 95.0%). (TIF) [file pone.0186287.s002.tif]

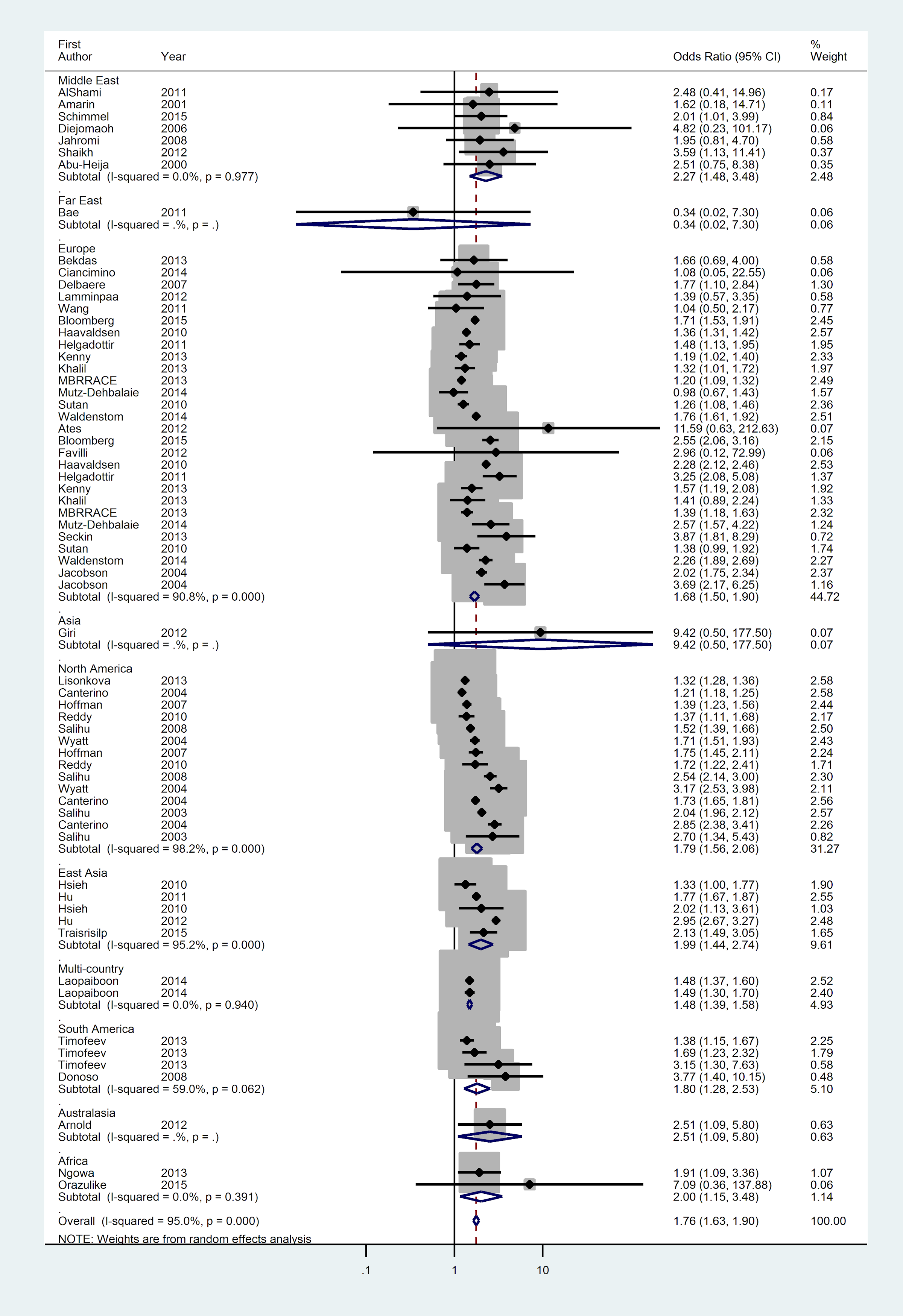

Supplement: S2 Fig — Heterogeneity was classified as severe for (Cochran’s χ2, I2 = 95.0%). (TIF) [file pone.0186287.s003.tif]

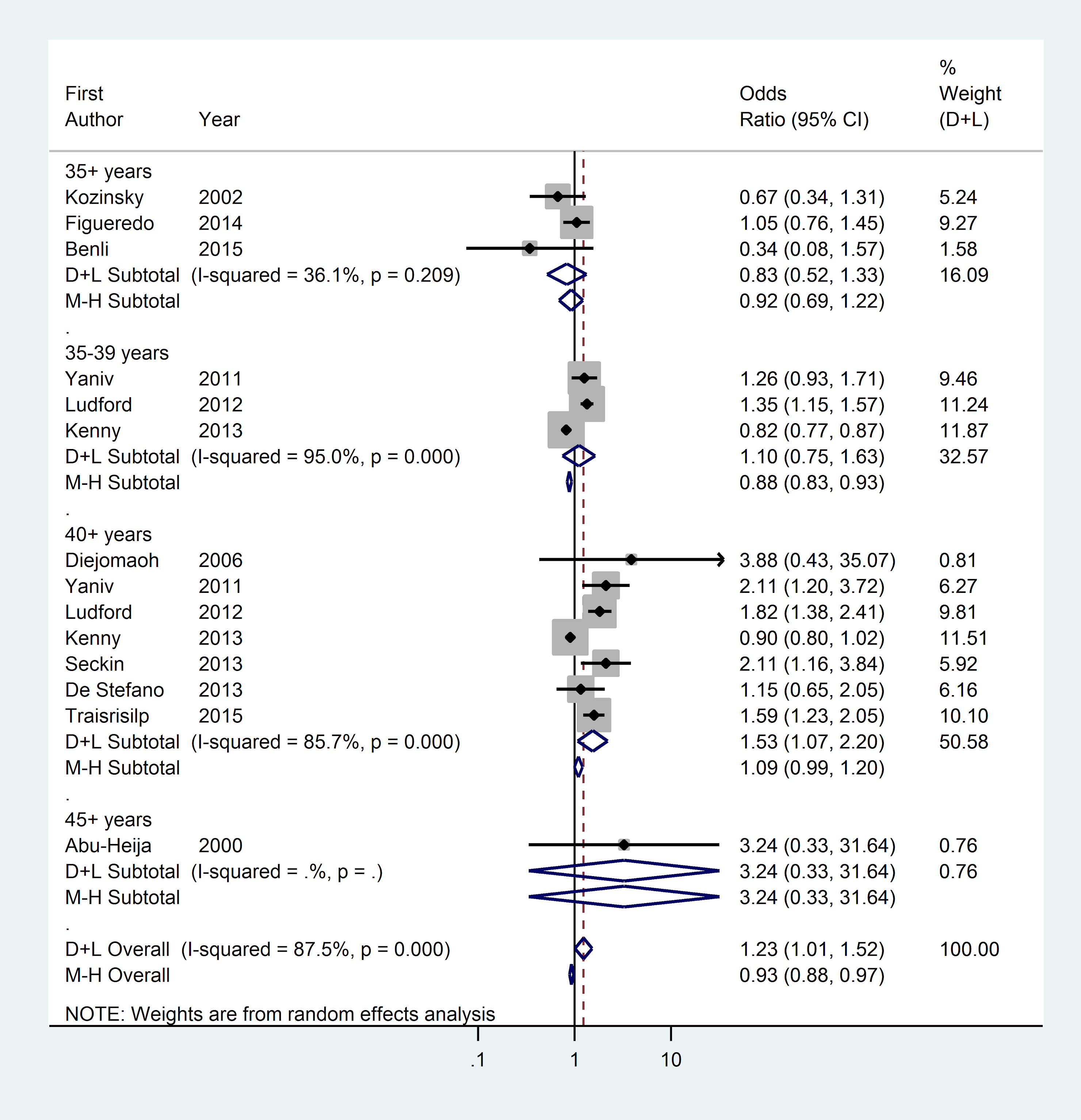

Supplement: S3 Fig — Heterogeneity was classified as severe (Cochran’s χ2, I2 = 87.5%). (TIF) [file pone.0186287.s004.tif]
