# Supplementary material for: Advanced maternal age and adverse pregnancy outcomes: A systematic review and meta-analysis
Source: PLoS One. 2017 Oct 17;12(10):e0186287. doi: 10.1371/journal.pone.0186287 (PMC5645107; doi:10.1371/journal.pone.0186287)
Supplement: S1 Appendix — (DOCX) [file pone.0186287.s005.docx]

**S1 Appendix**

**Search Strategy for Ovid®**

| #▲ | Searches | Results |
| --- | --- | --- |
| 1 | Maternal age.mp. [mp=ti, ot, ab, tx, ct, sh, kw, de, hw, bt, id, cc, nm, kf, px, rx, an, ui, tn, dm, mf, dv] | 77896 |
| 2 | Advanced.mp. [mp=ti, ot, ab, tx, ct, sh, kw, de, hw, bt, id, cc, nm, kf, px, rx, an, ui, tn, dm, mf, dv] | 1041410 |
| 3 | Pregnancy.mp. [mp=ti, ot, ab, tx, ct, sh, kw, de, hw, bt, id, cc, nm, kf, px, rx, an, ui, tn, dm, mf, dv] | 1980333 |
| 4 | Outcome.mp. [mp=ti, ot, ab, tx, ct, sh, kw, de, hw, bt, id, cc, nm, kf, px, rx, an, ui, tn, dm, mf, dv] | 3910288 |
| 5 | 1 and 2 and 3 and 4 | 2500 |
| 6 | limit 5 to yr="2000 -Current" [Limit not valid in DARE; records were retained] | 2124 |
| 7 | limit 6 to full text & cab abstracts fulltext [Limit not valid in Your Journals@Ovid,CDSR,ACP Journal Club,DARE,CCTR,CLCMR,CLHTA,CLEED,AMED,Econlit,GEOBASE,HMIC,MWIC,Ovid MEDLINE(R),Ovid MEDLINE(R) In-Process,Ovid MEDLINE(R) Daily Update,Ovid OLDMEDLINE(R),Embase; records were retained] | 2084 |
| 8 | limit 7 to english language [Limit not valid in Your Journals@Ovid,CDSR,ACP Journal Club,DARE,CLCMR,Econlit,HMIC,MWIC; records were retained] | 2018 |
| 9 | limit 8 to human [Limit not valid in Your Journals@Ovid,CDSR,ACP Journal Club,DARE,CCTR,CLCMR,AMED,CAB Abstracts,Econlit,GEOBASE,Global Health,HMIC,MWIC; records were retained] | 1939 |
| 10 | limit 9 to humans [Limit not valid in Your Journals@Ovid,CDSR,ACP Journal Club,DARE,CCTR,CLCMR,AMED,CAB Abstracts,Econlit,GEOBASE,Global Health,HMIC,MWIC; records were retained] | 1939 |
