# Supplementary material for: Advanced maternal age and adverse pregnancy outcomes: A systematic review and meta-analysis
Source: PLoS One. 2017 Oct 17;12(10):e0186287. doi: 10.1371/journal.pone.0186287 (PMC5645107; doi:10.1371/journal.pone.0186287)
Supplement: S2 Appendix — (DOCX) [file pone.0186287.s006.docx]

**Appendix 2**

## Systematic Review and Meta-Analysis Protocol

**Protocol: A Systematic Review and Meta-Analysis of Advanced Maternal Age and Adverse Pregnancy Outcomes**

**Background**

Advanced maternal age (AMA) is defined as childbearing in a woman over 35 years of age and is a growing trend within high-income countries (RCOG 2011). In 2013, 20% of births in England and Wales were to women aged 35 years or over and 4% to women ≥40 years compared to just 6% and 1% respectively in 1980 (ONS 2014). This trend is most commonly attributed to older primigravid women who delay childbearing by lifestyle choice or due to underlying subfertility, but also includes multiparous women continuing childbearing (Guedes and Canavarro 2014). Women in both groups have benefited from advancements in assisted reproductive technologies (ART). Although changes in social-economic circumstances and developments in ART have driven a shift towards childbearing later in life, this new trend creates a clinical risk. AMA is reported to be associated with a range of pregnancy complications including: fetal growth restriction (FGR), preeclampsia (PE), placental abruption, pre-term birth (PTB) and stillbirth by a series of epidemiological studies (Carolan 2013; Cleary-Goldman et al. 2005; De Stefano et al. 2013; Giri et al. 2012; Glasser et al. 2011; Jacobsson et al. 2004; Jahromi and Husseini 2008; Karabulut et al. 2013; Kenny et al. 2013; Khalil et al. 2013; Mehta et al. 2014; Odibo et al. 2006; Pasupathy et al. 2011; Reddy et al. 2006b; Salihu et al. 2008; Waldenstrom et al. 2014; Yogev et al. 2010). Systematic reviews and meta-analyses have demonstrated that AMA is associated with an increased risk of Caesarean birth (Bayrampour and Heaman 2010) and that AMA is a risk factor for stillbirth (Flenady et al. 2011; Huang et al. 2008). However, these latter studies have been limited by a lack of data regarding the causation of stillbirth in AMA pregnancies and have considered stillbirth in isolation. One structured review of pregnancy risks in women ≥45 years found increased rates of pre-existing hypertension and pregnancy complications, such as gestational diabetes mellitus (GDM), gestational hypertension and PE, which may predispose to stillbirth (Carolan 2013). However, further studies are needed to examine the relationship between AMA and adverse pregnancy outcomes and consider underlying causes for the reported increased risk of stillbirth in this population.

Placental dysfunction plays a key role in the aetiology of stillbirth, preeclampsia and FGR, but comparatively little is known about the underlying biological mechanisms and their relationship with AMA. Currently, a lack of understanding of the pathophysiology impairs effective clinical management for AMA mothers. Motions to monitor and improve their pregnancy outcomes could be investigated, while minimising unnecessary intervention. We hypothesise that AMA is associated with a range of underlying placental pathologies that contribute to the increased risk of stillbirth and perinatal death. This systematic review aims to: i) examine the evidence linking AMA and stillbirth, and ii) investigate whether AMA is also associated with other pregnancy complications resulting from placental dysfunction.

**Review question:** To determine the risk of stillbirth and other associated adverse pregnancy outcomes related to placental dysfunction in women of advanced maternal age (AMA, ≥35 years).

**PICOS** *(Population, Intervention (test), Comparison, Outcome, Setting):*

**P:** Adult pregnant females aged <35 and ≥35 years of age

**I:** Mothers aged ≥35 years at delivery

**C:** Mothers aged <35 years at delivery

**O**: Primary outcomes: Stillbirth (according to individual study gestational age cut off); FGR defined as birthweight below 5^th^ centile adjusted for gestational age. Undefined FGR will be included. FGR defined as below the 10^th^ centile will be classified as small for gestational age (SGA) baby

Secondary outcomes: Neonatal death (NND) according to individual study; SGA defined as birthweight below 10^th^ centile adjusted for gestational age. Undefined SGA was still included. SGA defined as below the 5^th^ centile was classified as FGR; Neonatal intensive care unit (NICU) admissions and neonatal acidosis; Preeclampsia; Placental abruption; Preterm birth (PTB) defined as birth <37 weeks gestation; Neonatal Acidosis (NNA) defined as umbilical artery pH <7.1

**S:** Studies from any countries reporting outcomes for all recorded births (in and outside the hospital setting)

**Inclusion criteria for studies:**

- Peer reviewed publications
- Human adult populations
- Full Text
- English language
- Year of Publication ≥2000

**Publication type:**

Peer reviewed published journals and papers and national perinatal reports. Case-control and cohort, studies performed prospectively and retrospectively.

**Study selection:**

Two researchers will screen titles and abstracts and then extract data from full papers.

**Data Extraction:**

Data extraction will be performed by the primary researcher, Samantha Lean, and will be checked by the Dr Hayley Derricott. Discrepancies and resolution will be noted and corrected. Disagreements will be resolved by discussion with a senior author (AH).

**Definitions:**

1. Maternal Age: Maternal age at delivery
2. Advanced: ≥ 35 years of age
3. Pregnancy: Current gestations during study period, singleton pregnancies
4. Outcome: Fetal Outcomes in pregnancy including: stillbirth, FGR, SGA, NND, NICU admissions, PE, PTB and NNA.

**Electronic Searches:**

1. MEDLINE (Ovid)
2. Embase
3. Cochrane database of Systematic Reviews
4. LILACS
5. ClinicalTrials.gov
6. National Perinatal Mortality Reports
7. Year of Publication ≥2000
8. English language
9. Keywords: Maternal Age AND advanced AND Pregnancy AND Outcome
10. Screen out ages below 18

**Searching Other Resources:**

Reference lists of generated articles will be screened to identify further relevant studies. Conference proceeding suitable to the authors will be screened for relevant abstracts.

**Selection of Studies:**

- 1. Content: Any quantitative design reporting pregnancy outcomes of interest in mother ≥35 years of age compared to a control young population (<35 years of age)
  2. Software: online searches and EndNote Reference Management
  3. Pilot: A pilot search was run in Medline to scope quality of search terms and quantity of tittles generated. Initial scope of themes and search content generated exclusion criteria for final search

**Process:**

Two review authors will independently assess for inclusion and exclusion criteria for all the potential studies we identify as a result of the search strategy. We will resolve any disagreement through discussion or, if required, will consult a third author (AH). We will create a study flow PRISMA diagram to map out the number of records identified, included and excluded. Two independent researchers will perform data extraction. Any discrepancies between researchers’ data extraction were clarified by primary researcher.

Following the above, the tool that will be used for data extraction is:

| Author | Year | Study Type | Place | Pregnancy Outcome | AMA Age | #AMA | Outcome Frequency | Control Age | #Control | Outcome Frequency |
| --- | --- | --- | --- | --- | --- | --- | --- | --- | --- | --- |
|  |  |  |  |  |  |  |  |  |  |  |

**Quality Assessment:**

Risk of Bias Assessment for Included studies: Due to methodological issues regarding quality assessment of observational studies in epidemiology, we will assess the quality of studies in eight specific domains that are relevant to the studies in question developed from Sanderson et al (Sanderson et al. 2007). This will be conducted by two authors independently.

| **Risk of Bias Assessment** | **Classification** | **Assessment** |
| --- | --- | --- |
| Were conflicts of interest disclosed in the manuscript? | Low/ High/  Unclear | Low = stated no conflicts of interest;  High = stated conflicts of interest;  Unclear = no statement regarding conflicts of interest was included. |
| Were appropriate statistical methods used to report primary analysis of effect? | Low/ High/  Unclear | Low – appropriate statistical test reported.  High – inappropriate statistical test reported.  Unclear = vague/general or not stated statistical tests |
| Were appropriate methods used to control for confounding factors? | Low/ High/  Unclear | Low – appropriate controls for confounder e.g. BMI/maternal disease.  High – did not control for any confounders  Unclear – did not state whether controlled for or not. |
| Were all outcome measures reported? | Low/ High/  Unclear | Low – results data existed for all outcomes that were stated as reported in methods  High – data lacking for an outcome they reported that they measured  Unclear – had vague or no description on outcomes they were going to record |
| Was outcome data complete? | Low/ High/  Unclear | Low – 98-100% completeness of records or excluding incomplete records from analysis  High – stated high levels of missing data from database/records  Unclear – completeness or data was not stated |
| Were the assessors blinded to group assignment (for case control studies)? | Low/ High/  Unclear | Low – assessor did not know the age of population groups at time of analysis  High – assessors were not blinded or there was no way to assess when blinded  Unclear – it was not clear if assessor was blinded or not |
| Method of measurement of outcome? | Low/ High/  Unclear | Low – standard clinical use, low inter-user variation, standard classification of outcomes used  High – high intra-user variability, did not specify how outcome measures were defined, not standard or un-optimised measurements performed  Unclear – Did not state method of measurement of outcomes or historical retrospective data analysed with methods unclear |
| Was there evidence of selection bias? E.g. only a specific group of participants studied | Low/ High/  Unclear | Low – large retrospective or cohort studies with few exclusion criteria  High – case control study where specific cases were included with no evidence of matching for controls  Unclear – Not clear on inclusion/exclusion criteria or participant/record selection process for study |

**Data Synthesis:**

1. Meta-analysis will be conducted using STATA (Version 13, StataCorp, Texas, USA) using the *metan*, *meta-regression* and *meta-bias* commands.
2. Random effects meta-analysis will be performed in view of the anticipated heterogeneity between the studies. Heterogeneity will be quantified using Cochran’s χ^2^ test to generate I^2^ statistic as a percentage of variability. Heterogeneity will be classified as low (I^2^= 0-40%), moderate (I^2^ = 30-60%), high (I^2^ = 50-90%) or severe (I^2^ = 80-100%; Cochrane, 2013).
3. Where possible, *meta-regression* will be undertaken to test the effect of maternal age as a linear variable.
4. To explore any heterogeneity Forest plots will be constructed to allow subgroup analysis of maternal age, geographical location and year of study on each outcome.
5. Funnel plots and contour enhanced funnel plots will be created to test for publication bias and small study effects quantified by application of Harbord’s test.
6. Population attributable risk (PAR) will be calculated for the proportion of stillbirths associated with AMA.

**Dissemination:**

The aim will be to submit for publication to a high impact general medical journal.
